# Validation of epidemiological tools for eczema diagnosis in brazilian children: the isaac's and uk working party's criteria

**DOI:** 10.1186/1471-5945-10-11

**Published:** 2010-11-09

**Authors:** Agostino Strina, Mauricio L Barreto, Sergio Cunha, Maria de Fátima SP de Oliveira, Shirlei C Moreira, Hywel C Williams, Laura C Rodrigues

**Affiliations:** 1 Universidade Federal da Bahia, Instituto de Saúde Coletiva, Salvador, Bahia, Brazil; 2 Universidade Federal de Pernambuco, Centro de Ciências da Saúde, Departamento de Medicina Social, Recife, Pernambuco, Brazil; 3 Universidade Federal da Bahia, Serviço de Dermatologia, Salvador, Bahia, Brazil; 4 Ministério da Saúde, Núcleo Estadual da Bahia, Salvador, Bahia, Brazil; 5 Queen's Medical Centre, Department of Dermatology, Nottingham, UK; 6 London School of Hygiene and Tropical Medicine, Epidemiology and Population Health, London, UK

## Abstract

**Background:**

Instruments for field diagnosis of eczema are increasingly used, and it is essential to understand specific limitations to make best use of their strengths. Our objective was to assess the validity of ISAAC and UK Working Party criteria for field diagnosis of eczema in children.

**Methods:**

We performed a cohort study in urban Brazil. Parents/guardians of 1,419 children answered ISAAC phase II questionnaire. Children were examined for skin lesions (UKWP protocol). Two dermatologists examined most cases of eczema (according to ISAAC or UKWP), and a sample without eczema.

**Results:**

Agreement between repeat questionnaires on the filter question was poor (kappa = 0.4). Agreement between the 2 dermatologists was fair (kappa = 0.6). False positive reports included scabies in 39% of ISAAC cases and 33% of UKWP cases. Sensitivity and PPV were low (ISAAC: 37.1% and 16.1%; UKWP: 28.6% and 23.8%). Specificity and NPV were high (ISAAC: 90.0% and 96.6%; UKWP: 95.3% and 96.2%). One-year prevalence of eczema was 11.3% (ISAAC), 5.9% (UKWP) and 4.9% (adjusted dermatologist diagnosis). Point prevalence of scabies (alone or not) was 43%, 33% and 18%, in eczemas according to ISAAC, to UKWP and to dermatologists. The reasons why children with eczema were not identified by ISAAC or UKWP were wrongly denying dry skin, itchy rash or personal history of atopic diseases. A limitation is that questionnaire was already validated in Brazil, but not field tested in this specific setting.

**Conclusions:**

Studies using UKWP or ISAAC criteria should include a validation arm, to contribute to the understanding of potential limitations of their use in different contexts and to explore solutions. We list specific recommendations.

## Background

Eczema has recently been proposed by the World Allergy Organization [1] to replace terms such as atopic dermatitis, atopic eczema, atopic eczema/dermatitis syndrome, previously used interchangeably in the literature [2]. Its individual diagnosis is a relatively undisputed matter: a chronic, or chronically relapsing, inflammatory skin disease, characterized by itchy papules, which become excoriated and lichenified, whose distribution pattern changes during lifetime from more generalized eruptions with oozing and crusted lesions in early childhood, to the childhood pattern of typical flexural eczema with lichenification and a scaly, xerotic, uninvolved skin, to the adult distribution pattern, essentially similar to that in later childhood [3]. However, the complexities of the clinical aspects of eczema, even after the pioneer effort by Hanifin and Rajka to organize them in a set of diagnostic elements [4], make them unsuitable for use in population-based studies. Other groups set more recently about establishing standardized criteria for eczema diagnosis in population settings [5-7]. Some elements of the protocol proposed by Williams et al (the so called UK Working Party (UKWP) criteria), which combines a standardized questionnaire and a set of reference photographs, were incorporated in the instrument (a questionnaire) adopted by the International Study of Asthma and Allergies in Childhood (ISAAC) [8] in its surveys.

Validation studies of ISAAC and UKWP criteria, mostly conducted in children and young subjects, found variable results [9-22]. Some studies, mainly carried out in developed countries, found high sensitivity and specificity [9,10,14,16-18,22], whereas others, mainly in low and middle income countries, found middle to low sensitivity and high specificity [11-13,15,19,20]. Two recent studies in adults, both in a developed country, found middle to low sensitivity and high specificity [23,24]. The reasons behind the variations are not clear, but the following suggestions have been put forward: intermittent nature of eczema [22], mild forms of eczema in the community (compared to those in hospitals or clinics), high prevalence of scabies (compared to the prevalence of eczema) [20], recall bias [24], language misunderstanding or cultural specificities [12,15,19,22], in particular denial of itching from parents [14,20], leading to false negative diagnosis. A recent review lists eight groups of possible reasons for the variation in validity: differences in study characteristics, different reference standards, different periods of prevalence to establish the diagnosis of eczema, high prevalence of scabies, exclusion of "visible flexural dermatitis" from the criteria, translation and cultural issues (such as the interpretation of pruritus), low prevalence of eczema and different methodological strengths [25].

This study aimed to validate the ISAAC and UKWP coding of the UKWP-ISAAC questionnaire for eczema in an urban setting of a middle developed country.

## Methods

This study represents the eczema arm of a cohort study conducted in the city of Salvador, Brazil, to study allergic diseases. The design of the original study is presented in detail elsewhere [26]. The methods relevant for this analysis are presented below.

**Figure 1:**
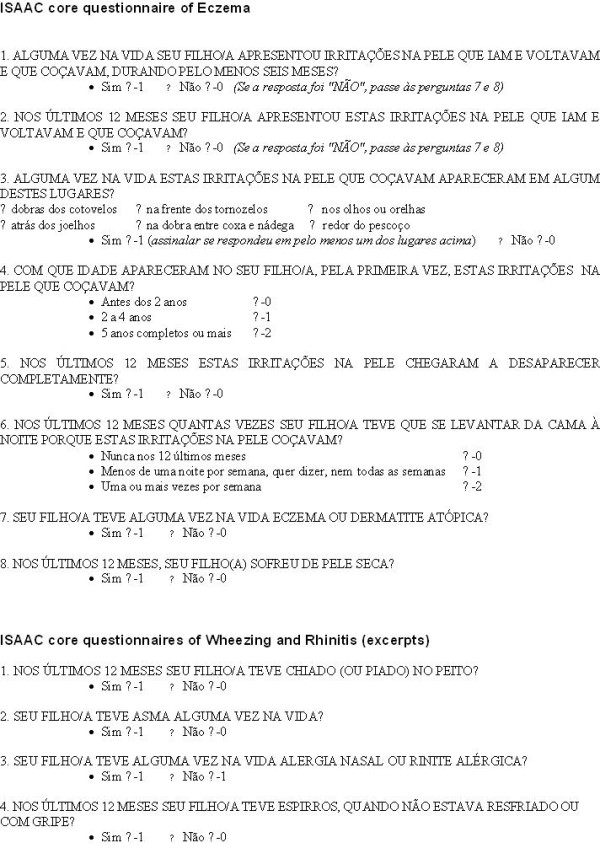
**Field Questionnaire**.

The study population consisted of 1,419 children. Their parent\guardians answered the ISAAC phase II core questionnaire for eczema [24], translated into Portuguese and back-translated into English, and validated [18]. The relevant questions are presented in Figure 1. The children were also examined for skin lesions according to the UKWP protocol. Because of the overlap between slightly different filter questions in ISAAC and UKWP questionnaires, we used only the ISAAC version so that the first two questions of the ISAAC core questionnaire ("Has your child ever had an itchy rash that came and went for at least 6 months?" and "Has your child had this itchy rash at any time in the last 12 months?") were used as filter questions for the UKWP protocol too, in place of the original one ("Has your child had an itchy skin condition in the last 12 months?"). So we departed from the recommended UKWP protocol only in that we included in the screening questions a requirement for the itchy lesion to come and go for at least 6 months rather than just having been present.

Two trained observers examined the children and applied the questionnaire. The observers, two health operators with auxiliary nurse training, spent some sessions in an outpatient clinic with a dermatologist, who introduced them to the types of basic skin lesions and showed them children with different skin problems including eczema, briefly commenting the characteristics of the lesions (aspect, site and history). Besides seeing patients, the observers were trained to recognize the flexural dermatitis through the use of the UKWP photographic protocol and training photographs set. All children, not only those with a report of recurrent itchy skin lesions, were examined. Results of the physical examination were noted in terms of whether there was a skin lesion, what type, in what body area, and whether it was consistent with any of the 20 UKWP photographs.

Data from the questionnaire were coded by one of the authors (AS). Cases were defined based on two coding systems:

1) ISAAC criteria (positive responses to the questions 1 to 3 of the core questionnaire of eczema, Figure 1) [27];

2) UKWP criteria (positive response to question 2, plus three or more of the following supplementary elements: history of flexural involvement (question 3), onset below 2 years of age (question 4), history of generally dry skin (question 8), personal history of other atopic disease, and visible flexural dermatitis as per photographic protocol) [24]. The history of other atopic diseases was based on questions from the ISAAC's core questionnaires of wheezing and rhinitis, Figure 1, i.e., a positive response to any question from 1 to 3 or a positive response to question 4 plus reported use of antihistaminic drugs and/or topic corticosteroids. The field workers, the parent/caretakers and the dermatologists remained blind to the classification.

For operational reasons, the questionnaires were applied twice to about half the children (736); these were used to estimate repeatability. Data were collected between September 2005 and March 2006.

A sample of children was independently seen by 2 dermatologists for validation. Children were selected for referral based on their answers to the ISAAC questionnaire (for children interviewed twice, the second questionnaire was used). Children were classified in three groups, with different sampling frames for referral. The first group consisted of children with eczema according to either ISAAC or UKWP. The second group consisted of children without eczema according to ISAAC or UKWP, but with a positive response to the question: "Has your child had this itchy rash that came and went at any time in the past 12 months?". The third group consisted of children without eczema or itchy rash. The proportions selected for validation in each group were roughly: 100% of the first group, half of the second group, and one in twenty of the third group.

The proportions seen by the dermatologists in each group are presented in Table 1. The dermatologists, both of which were consultant dermatologists, conducted independently a clinical consultation to establish if the child had eczema based on history and clinical examination, and prepared a standard report that included whether in their opinion the child had eczema (as eczema, possible eczema and no eczema) and any alternative diagnosis.

**Table 1:** Children seen in the field and visited by the dermatologists

	seen in the field	seen by dermatologists	sampling fraction (SF)	weighting (1/SF)
ISAAC_+_	161	150	93.2%	1.07
ISAAC- scratchers_+_	51	25	49.0%	2.04
ISAAC- scratchers-	1207	58	4.8%	20.81
UKWP_+_	84	78	92.9%	1.08
UKWP- scratchers_+_	128	97	75.8%	1.32
UKWP- scratchers-	1207	58	4.8%	20.81

"scratcher' by filter question: "Has your child had this itchy rash that came and went at any time in the past 12 months?"

For the children with repeated questionnaires we investigated agreement between first and second questionnaires for the filter question ("Has your child ever had an itchy rash that came and went for at least 6 months?"), and for the diagnosis of eczema using UKWP and ISAAC criteria separately, according to whether they had the same respondent and the same observer.

After examining the consistency between the two dermatologists, children classified by them as possible eczemas were grouped with those classified as non eczema. Agreement between the diagnoses of the two dermatologists was assessed by contingency tables and kappa statistics.

To examine the reasons for disagreement between the diagnosis based on the questionnaire and made by the dermatologists, we considered as the gold standard for eczema a diagnosis of eczema by at least one dermatologist. The dermatologists' diagnoses for false positives were listed and the reasons for false negatives were investigated.

Due to different fractions having been sampled for referral from each of the groups (children with eczema, without eczema but with an itchy rash, and without eczema or itchy rash), the prevalence of eczema PR was independently calculated, for each of the two field definitions of eczema, on the original population weighted by the inverse of the sampling fraction of each group (Table 1), according to the formula PR = (E(dr)_Gr1_*W_Gr1 _+ E(dr)_Gr2_*W_Gr2 _+ E(dr)_Gr3_*W_Gr3_)/Pop*100, where E(dr) is the number of eczemas diagnosed by the dermatologists in a group and W its weighting, and Pop is the total population investigated.

Using dermatologist diagnosis as a gold standard, sensitivity, specificity, positive predictive value (PPV) and negative predictive value (NPV) were estimated separately for ISAAC and UKWP coding. To estimate the potential improvement in UKWP validity if photographs had been more widely used, we recalculated sensitivity, specificity, PPV and NPV if the 8 false negative cases had had a positive photo identification.

Contingency tables, sensitivity, specificity, PPV, NPV and kappa statistics were calculated with Stata (ver.9.0).

Ethical approval was obtained from the LSHTM and from the UFBA ethical committee. A written informed consent was obtained from the parents of the children involved.

## Results

Of the 1,445 eligible children, 1,419 (758 boys), aged between 4 and 12 (median 7, IQR 6-9) years, completed the questionnaire and were included in the study.

The median interval between first and second questionnaire was 4.4 months. The agreement between the first and the second questionnaires for the filter question ("have you ever had an itchy rash...") was poor (kappa = 0.33) for all the pairs, and only slightly better for the pairs with the same respondent, and those with the same respondent and observer (Table 2). This was mainly because of the high proportion of positive answers in the first questionnaire that were negative in the second questionnaire (53%). This was very marginally better when the respondent was the same (48%) and the respondent and observer were the same (47%)

**Table 2:** Consistency between 1^st ^and 2^nd ^questionnaire to the ISAAC filter question^1^

	all the pairs			same respondent			same respondent and same observer		
**1^st ^questionnaire**	**2nd questionnaire**			**2nd questionnaire**			**2nd questionnaire**		
	**yes**	**no**	**Total**	**yes**	**no**	**Total**	**yes**	**no**	**Total**
yes	97	108	205	78	71	149	46	41	87
no	70	367	437	49	267	316	27	130	157
Total	167	475	642	127	338	465	73	171	244
	*kappa = 0.33* *95% CI 0.25-0.41*			*kappa = 0.38* *95% CI 0.29-0.47*			*kappa = 0.37* *95% CI 0.25-0.49*		

^1^"Has your child ever had an itchy rash that came and went for at least 6 months?"

The agreement between the first and the second questionnaires on presence of eczema was poor for all pairs (kappa = 0.34, when coded using ISAAC, and kappa = 0.29, using UKWP). When kappa was calculated separately for pairs with the same respondent and the same respondent and observer, there was a slight improvement for ISAAC coding, and a more marked improvement for UKWP coding. Once again, the poor agreement was mainly attributable to the high proportion of children classified as having eczemas in the first questionnaire that were not classified as eczema in the second questionnaire (64% for ISAAC coding, and 74% for UKWP coding). These proportions were smaller, but still over half, when the respondent was the same (56% for ISAAC and 61% for UKWP) and when the respondent and the observer were the same (58% for ISAAC and 63% for UKWP) (Table 3).

**Table 3:** Consistency of diagnosis of eczema between 1^st ^and 2^nd ^questionnaire

	all pairs			same respondent			same respondent and same observer		
			
ISAAC coding 1^st ^questionnaire	2^nd ^questionnaire			2^nd ^questionnaire			2^nd ^questionnaire		
			
	Yes	No	Total	Yes	No	Total	Yes	No	Total
Yes	33	58	91	28	35	63	14	19	33
No	34	515	549	27	373	400	14	195	209
Total	67	573	640	55	408	463	28	214	242
	*kappa = 0.34* *95% CI 0.26-0.41*			*kappa = 0.40* *95% CI 0.31-0.49*			*kappa = 0.38* *95% CI 0.26-0.51*		
**UKWP coding** **1^st ^questionnaire**	**2^nd ^questionnaire**			**2^nd ^questionnaire**			**2^nd ^questionnaire**		
			
	Yes	No	Total	Yes	No	Total	Yes	No	Total
Yes	12	28	40	12	19	31	7	12	209
No	21	579	600	13	419	431	4	219	33
Total	33	607	640	25	438	463	11	231	242
			
	*kappa = 0.29* *95% CI 0.21-0.37*			*kappa = 0.39* *95% CI 0.30-0.48*			*kappa = 0.43* *95% CI 0.31-0.56*		

Of the 233 children referred to the dermatologists, 212 were seen by both dermatologists. The median time between field and validation was 8 days (IQ range 6-21.5 days) Agreement between dermatologists was fairly good for non-eczemas (89.8% and 88.8% of non-eczemas according to one dermatologist were non-eczemas for the other one). Agreement was less good for eczema diagnosis (63.2% and 62.3% of eczemas according to one dermatologist were eczemas for the other one), and was very poor for the diagnosis of possible eczema, most of which (82.4% and 86.7%) were not eczemas for the other dermatologist (Table 4). We decided, therefore, to group non-eczemas and possible eczemas in the same category for each dermatologist. After this, the kappa for concordance between the two dermatologists was 0.56.

**Table 4:** Consistency of diagnosis between the 2 dermatologists

	**2^nd ^dermatologist's diagnosis**			
	
**1^st ^dermatologist's diagnosis**	**eczema _+_**	**possible** **eczema**	**eczema -**	**Total**
	
eczema _+_	12	1	6	19
possible eczema	2	1	14	17
eczema -	5	13	158	176
Total	19	151	171	212

kappa = 0.56 (95% CI 0.46-0.73), with negative and possible cases collapsed together

We examined the reports for children with a false positive field diagnosis (129 children by ISAAC and 59 by UKWP). Each child had a maximum of two reports, one from each dermatologist. The proportions of ISAAC false positives reports and UKWP false positives reports that included scabies were 39% and 33%, respectively, that included prurigo were 12% (ISAAC) and 15% (UKWP) and with no dermatologic alteration were 10% (ISAAC) and 13% (UKWP). Other diagnoses were present in smaller numbers (Table 5).

**Table 5:** Dermatologists' diagnoses for children with eczema according to ISAAC or UK Working Party, but not according to dermatologists

	**ISAAC** **eczemas** **(N = 126)**	**UKWP** **eczemas** **(N = 59)**
		
Scabies	39.0%	33.3%
prurigo (strophulus)	11.6%	14.9%
no dermatologic alteration	10.4%	13.2%
miliaria (prickly heat)	5.4%	7.0%
residual hypercromic macules	4.1%	3.5%
pityriasis alba	3.7%	3.5%
xerodermia	2.5%	5.3%
tinea corporis	2.5%	3.5%
dyshidrotic eczema	2.5%	2.6%
others	18.3%	13.2%

Table 6 shows the proportion of children who had a diagnosis of scabies (alone or in combination with another diagnosis) by at least one of the dermatologists, separately for ISAAC and UKWP coding, in each of six categories, i.e., two of dermatologist diagnosis of eczema by three of field assessment (eczema and non-eczema with or without an itchy rash). The prevalence of scabies, as diagnosed by dermatologists, was high (36%). Among children with no reported itching in the questionnaire, 19% had a diagnosis of scabies. The proportion with scabies was higher in children with a diagnosis of eczema according to ISAAC (43%) than in those diagnosed with eczema according to UKWP (33%). Only 18% of the children diagnosed as eczema by the dermatologists had scabies.

**Table 6:** Frequency of scabies diagnosis (made by any dermatologist)^1^, by field assessment and dermatologist's diagnosis of eczema

	Dermatologists' diagnosis								
	
	Eczema_+_			eczema-			Total		
		
ISAAC									
eczema_+_	24	*5*	*20.8%*	126	*59*	*46.8%*	150	*64*	*42.7%*
eczema-/scratcher_+_	1	*0*	*0.0%*	24	*8*	*33.3%*	25	*8*	*32.0%*
eczema-/scratcher-	2	*0*	*0.0%*	56	*11*	*19.6%*	58	*11*	*19.0%*
UKWP									
eczema_+_	19	*2*	*10.5%*	59	*24*	*40.7%*	78	*26*	*33.3%*
eczema-/scratcher_+_	6	*3*	*50.0%*	91	*43*	*47.3%*	97	*46*	*46.4%*
eczema-/scratcher-	2	*0*	*0.0%*	56	*11*	*19.6%*	58	*11*	*19.0%*

Total	27	*5*	*18.5%*	206	*78*	*37.9%*	233	*83*	*35.6%*

^1^Number and percentage of scabies diagnosis in italic

'scratcher' by filter question: "Has your child had this itchy rash that came and went at any time in the past 12 months?"

When dermatologists diagnosed eczema in children coded as non-eczema by ISAAC or UKWP, the reasons for the disagreement were: the caretaker denied the child suffered from dry skin in the last 12 months but the dermatologist found dry skin (in 1 non-eczema according to ISAAC and 5 according to UKWP); the caretaker denied the presence of an itchy rash (ever or in the past 12 months), but the dermatologist elicited its presence in the child's history (in 2 non-eczemas according to both ISAAC and UKWP); the caretaker denied the personal history of asthma/rhinitis, but the dermatologist elicited such a history, in 1 non-eczema according to UKWP. Four of the 8 false negatives according to UKWP would have been coded as eczema if they had had a positive photograph identification.

The prevalence estimated using the questionnaire is very different when coded to ISAAC (11.3%) and to UKWP (5.9%). Applying the weights from the sampling fractions from Table 1 to the dermatologist diagnosis, the estimated true prevalence of eczema in the original population (using either ISAAC or UKWP) was 4.9%. We estimated the validity of each coding scheme using the dermatologist diagnosis as the gold standard, and using the weights from the sampling fraction. The sensitivity, specificity, PPV and NPV were, for ISAAC, 37.1%, 90.0%, 16.1% and 96.6%, respectively, and, for UKWP, 28.6%, 95.3%, 23.8% and 96.2%, respectively. The UKWP sensitivity and PPV would have been a little higher if the false positives had had a positive photograph identification (35.7% and 28.4%), but the specificity and NPV would have been very similar (96.0% and 96.6%). The agreement between dermatologists diagnosis and ISAAC field diagnosis was very low (kappa = 0.09), and between dermatologists and UKWP field diagnosis was low (kappa = 0.23).

At the physical examination by the field workers, 1,142 children overall had skin lesions, 433 had lesions in flexural sites, 217 had lesions compatible with eczema following the UKWP criteria (scaling, vesicles, crusts or lichenification, whether or not associated with erythema) [28], and 105 had lesions compatible with eczema in a flexural area. Only 32 children had lesions that were linked to a photograph in the UKWP protocol.

Of the 105 children who presented lesions compatible with dermatitis in a flexural area, only 13 were classified as eczema by the UKWP criteria. Of the 92 that were not, 66 replied no to the first filter question ("Has your child ever had an itchy rash that came and went for at least 6 months?") and an additional 9 no to the second filter question ("Has your child had this itchy rash at any time in the last 12 months?"). As for the 17 left, the carer referred for most of them a history of itchy lesions in flexural sites in the past 12 months, but very rarely a history of dry skin or a personal history of other atopic diseases, and the field worker did not associate any photo of the UKWP protocol. Of the 105 children with lesions compatible with dermatitis in a flexural area, 31 were seen by the dermatologists for validation. Of the 31 children, 12 had eczema according the UKWP criteria. Of these 12, 1 was diagnosed by the dermatologists as having eczema, 6 as having scabies, and the other 5 with a range of other diagnoses, including miliaria and prurigo.

## Discussion

In this validation study, UKWP coding was as good as ISAAC on specificity and NPV, and better, but still not very good, on sensitivity and PPV. Agreement between first and second questionnaire was not good, even when observer and respondent were the same. False positives were mostly attributable to scabies (mostly for ISAAC coded questionnaires) but since specificity was high, this was not the main problem. False negatives were mostly caused by respondents denying itch, dry skin or a history of allergic diseases when those features were identified by the dermatologists on their validation. Although observers identified and described lesions in the majority of the children, they were very reluctant to link lesions to photographs. Had the false negatives been linked to a photograph, given the scoring scheme, half would have been considered eczema according to UKWP coding scheme. Recognition of a lesion compatible with eczema in a flexural site was rarely associated with UKWP diagnosis or with an eczema diagnosis by the dermatologists. Prevalence estimated by UKWP was quite accurate and very similar to that estimated based on doctors diagnosis, in spite of the low sensitivity. This was due to the fact that there were similar number of false positives and false negatives in the population. The ISAAC's prevalence estimate performed far less accurately, thus probably challenging, at least in this particular setting, its validity at the population level [29]. So in summary UKWP was better than ISAAC coding, but sensitivity was still low, although estimates of prevalence were accurate and specificity high.

The study had some limitations. The use of a more restrictive filter question than the usual UKWP criteria to allow evaluation of the ISAAC criteria could have potentially contributed to the low sensitivity. The use of this question was evaluated in the UK [7] and in that context did not lower sensitivity. However this is likely to be different in our setting where the low concordance for this question in the repeat sample suggests that this question was not well understood. This is particularly relevant because we did not field test the questionnaire in our setting, as it had been validated in Brazil earlier [18], nor did we use qualitative studies to investigate the social acceptability of reporting itching.

The observers linked lesions to the UKWP photos in very few children. We did not test the observers formally for their use of the photographs before going to the field. Had we done that, we might have extended their already substantial training,

In places where other skin diseases like scabies are common, the value of flexural dermatitis as diagnosis of eczema is limited. We suggest that it may be important to consider ways of dealing with scabies (maybe using response to treatment as a diagnostic test). This will be further complicated by the fact that in populations with high prevalence of scabies, people with eczema will also have scabies. Improved recognition of scabies however will improve specificity, not sensitivity.

The difficulty of identifying eczema during remission is well established. This might complicate validation studies if the dermatologists do not examine the children at the same time as the field observers. This is unlikely to have contributed to the disagreement between field diagnosis and the dermatologist, as the median time between field and validation was 8 days. Another potential limitation of validations is difference in duration of the period considered, for example when prevalence is measured over a 12 month period in the instruments and over a point in time by the dermatologists. In our study this was mitigated by the fact that the dermatologists were considering the long term diagnosis as they would in their clinical practice, based on history and examination,

Finally, the age of the study population was one where eczema prevalence is usually low. The children in our study were all aged over 4, and some were as old as 12. This is likely to be a problem when the ISAAC questionnaire is applied mostly to study asthma, and the eczema questionnaire used as a secondary objective. It is because of low prevalence that PPV is low in spite of the high specificity and NPV is high in spite of the low sensitivity. This is one more reason why validity estimated in hospital cases is not generalizable to situations when questionnaires are applied to a general population. As a consequence of the very low PPV most cases diagnosed in the field questionnaire are not eczema (are false positives) and therefore are not suitable for studies of causality.

Many of our findings are similar to previous validation studies, where the specificity of UKWP tended to be high, although the sensitivity was very varied. Low sensitivity in previous studies has been attributed to similar reasons as in our study.

## Conclusions

UKWP was better than ISAAC and less affected by scabies. Both had good specificity and low sensitivity, and UKWP was very accurate in estimating prevalence although this was based on similar numbers of false negative and false positive cases.

Our recommendations for studies of eczema using field instruments are that

1. Studies conducted in a new setting should field test the instrument even when it has been previously validated in another setting in the same country. The field test must aim to arrive at a form of word such that questions are clearly understood by the population, as denial in the questionnaire of the filter questions and of itching, dry skin and family history are behind the low sensitivity. It might be necessary to study whether there are cultural barriers to acknowledging itching.

2. Keep in mind that validity obtained using hospital cases may overestimate validity in the population.

3. Studies in which UKWP photographic protocol is employed always test the trained personnel in the use of photographs.

4. Where other itchy eczematous looking skin diseases (such as scabies) are common, the survey should train observers to recognize them to increase specificity.

In addition, our recommendations for validation studies of field instruments for diagnosis of eczema are that:

1. The time between field testing and validation be as short as possible.

2. The period over which eczema is diagnosed (point prevalence, one-year prevalence etc.) be the same for the field instrument and the gold standard.

3. Doctors used as the gold standard be as blind as possible to the field criteria.

4. Examine in detail false negatives and false positives to identify local obstacles to validity.

Finally, we believe that it is very important to continue to accumulate evidence on the potential limitations of the use of field instruments for the diagnosis of eczema and relevant solutions, and suggest that all studies using UKWP or ISAAC for research should include a validation arm.

## Abbreviations

ISAAC: International Study of Asthma and Allergies in Childhood; UKWP: United Kingdom Working Party; PPV: positive predictive value; NPV: negative predictive value.

## Competing interests

The authors declare that they have no competing interests.

## Authors' contributions

AS coordinated the survey, performed the interpretation and analysis of the data and drafted the manuscript; MLB and LCR participated in the study design and in the analysis and interpretation of data, and contributed to the manuscript drafting; SSC designed the study and participated in manuscript drafting; HCW contributed to the analysis, interpretation and drafting of the manuscript; MdeFSP and SCM provided the gold standard and participated in the manuscript drafting. All authors read and approved the final manuscript.

## Pre-publication history

The pre-publication history for this paper can be accessed here:

http://www.biomedcentral.com/1471-5945/10/11/prepub
